# Spindle oscillations in communicating axons within a reconstituted hippocampal formation are strongest in CA3 without thalamus

**DOI:** 10.1038/s41598-024-58002-0

**Published:** 2024-04-10

**Authors:** Mengke Wang, Samuel B. Lassers, Yash S. Vakilna, Bryce A. Mander, William C. Tang, Gregory J. Brewer

**Affiliations:** 1 Department of Biomedical Engineering, University of California, Irvine, CA 92697 USA; 2 Center for Neurobiology of Learning and Memory and MIND Center, University of California, Irvine, CA 92697 USA; 3 Institute for Memory Impairments and Neurological Disorders, University of California, Irvine, CA 92697 USA; 4 Texas Institute of Restorative Neurotechnologies (TIRN), The University of Texas Health Science Center (UTHealth), Houston, TX 77030 USA; 5 Department of Psychiatry and Human Behavior, University of California, Irvine, CA 92868 USA

**Keywords:** Spindle, LFP, Axon, Oscillation, EEG waves, Hippocampus, Entorhinal, Dentate, CA3, CA1, Axons, Neuroscience, Circadian rhythms and sleep, Neural circuits

## Abstract

Spindle-shaped waves of oscillations emerge in EEG scalp recordings during human and rodent non-REM sleep. The association of these 10–16 Hz oscillations with events during prior wakefulness suggests a role in memory consolidation. Human and rodent depth electrodes in the brain record strong spindles throughout the cortex and hippocampus, with possible origins in the thalamus. However, the source and targets of the spindle oscillations from the hippocampus are unclear. Here, we employed an in vitro reconstruction of four subregions of the hippocampal formation with separate microfluidic tunnels for single axon communication between subregions assembled on top of a microelectrode array. We recorded spontaneous 400–1000 ms long spindle waves at 10–16 Hz in single axons passing between subregions as well as from individual neurons in those subregions. Spindles were nested within slow waves. The highest amplitudes and most frequent occurrence suggest origins in CA3 neurons that send feed-forward axons into CA1 and feedback axons into DG. Spindles had 50–70% slower conduction velocities than spikes and were not phase-locked to spikes suggesting that spindle mechanisms are independent of action potentials. Therefore, consolidation of declarative-cognitive memories in the hippocampus may be separate from the more easily accessible consolidation of memories related to thalamic motor function.

## Introduction

Sleep spindles are prominent 10–16 Hz oscillations in EEG recordings that rise and fall in amplitude over 1–2 s during non-REM sleep^1^. A role in consolidation of memory is supported by evidence across multiple model systems, including those demonstrating correlations between more sleep spindles and better declarative memories^2^ and transcranial alternating current (tACS) modulation of visual memory^3^. In human subjects, reactivation of learning events during sleep showed correlations of performance of pattern separation with sleep spindle density^4^ and phase-dependent prefrontal cortical stimulation during sleep improved image recall the next day^5^. However, a role for spindles in the hippocampus remains unclear since other human studies with depth electrodes in surgical subjects found a predominance of non-communicating, isolated spindle oscillations^6^, which could be a sampling problem. The origin of sleep spindles was originally proposed to occur in the thalamic reticular nucleus^7^, based largely on their presence in extracellular recordings in isolated thalamic reticular nucleus from decorticated cat brain slices^8,9^. However, later investigations of the rat hippocampus in vivo indicated strong slow waves as well, in phase with cortical oscillations^10^. Andrade et al.^11^ found strong hippocampal fMRI signals associated with EEG sleep spindles in healthy human subjects, which could be endogenously generated or originate from the thalamus. Although Ngo et al.^12^ found clear spindle activity in the human hippocampus during N2 sleep, the activity was coincident in the hippocampus and neocortex, suggesting a third driving source or at least a common cue. By simultaneously recording local field potentials (LFP) and spikes in layers of the rat hippocampus and entorhinal cortex (EC), spiking in principal neurons did not correlate with particular phases of spindle oscillations despite strong current source density for the spindles in CA1 and DG^10^. These results raise the question of whether complex spike patterns on these neurons evoke spindle oscillations from intervening inhibitory neurons or whether spindles independently arise from axonal inputs.

In invasive recordings from human neurosurgical subjects, depth electrodes in the hippocampus and prefrontal cortex found a bidirectional relationship of information transfer between these regions during non-REM sleep^13^. They found spindle oscillations correlated with slow waves (0.16–1.5 Hz) that bidirectionally coupled hippocampal to prefrontal cortex, possibly evoking fast ripples (80–120 Hz) in the hippocampus. Cox et al.^14^ also studied human neurosurgical patients with implanted depth electrodes and at multiple frequencies and phase relationships to determine directionality of hippocampal coupling to neocortex. Although they found robust coupling of slow wave (0.5–1 Hz) and theta (6–9 Hz) oscillations from the hippocampus to the neocortex and strong spindle waves (9–15 Hz) within each structure, evidence was not found for phase-amplitude coupling of spindle oscillations from the hippocampus to the neocortex during N2 sleep or other stages. These findings raise important questions about the hippocampal origin of spindle oscillations in relation to the neocortex and whether they originate in the thalamus or the hippocampus.

With large 100 µm diameter depth electrodes, it is difficult to record the current–voltage changes in individual 30 µm neuronal somata, much less 0.1–0.5 µm diameter axons. This raises the important question whether spindle oscillations travel by volume conduction or axonal conduction as the mechanism for inter-regional communication. Clearly the signals that reach the scalp for EEG recordings get there by volume conduction even through the cranium, possibly from fields that emanate from bundles of axons in different regions of underlying cortex. Extracellular recordings in a milieu of brain neurons measure LFP in the surrounding vicinity. The source of the LFP is often assumed to be the voltage associated with the coordinate post-synaptic currents without actual measurement of those currents. However, these currents could also arise from parallel axons. Here we find that individual axons produce membrane potential changes without volume conduction, suggesting that the origin of spindle LFP may arise from responses to afferent axons.

**Figure 1:**
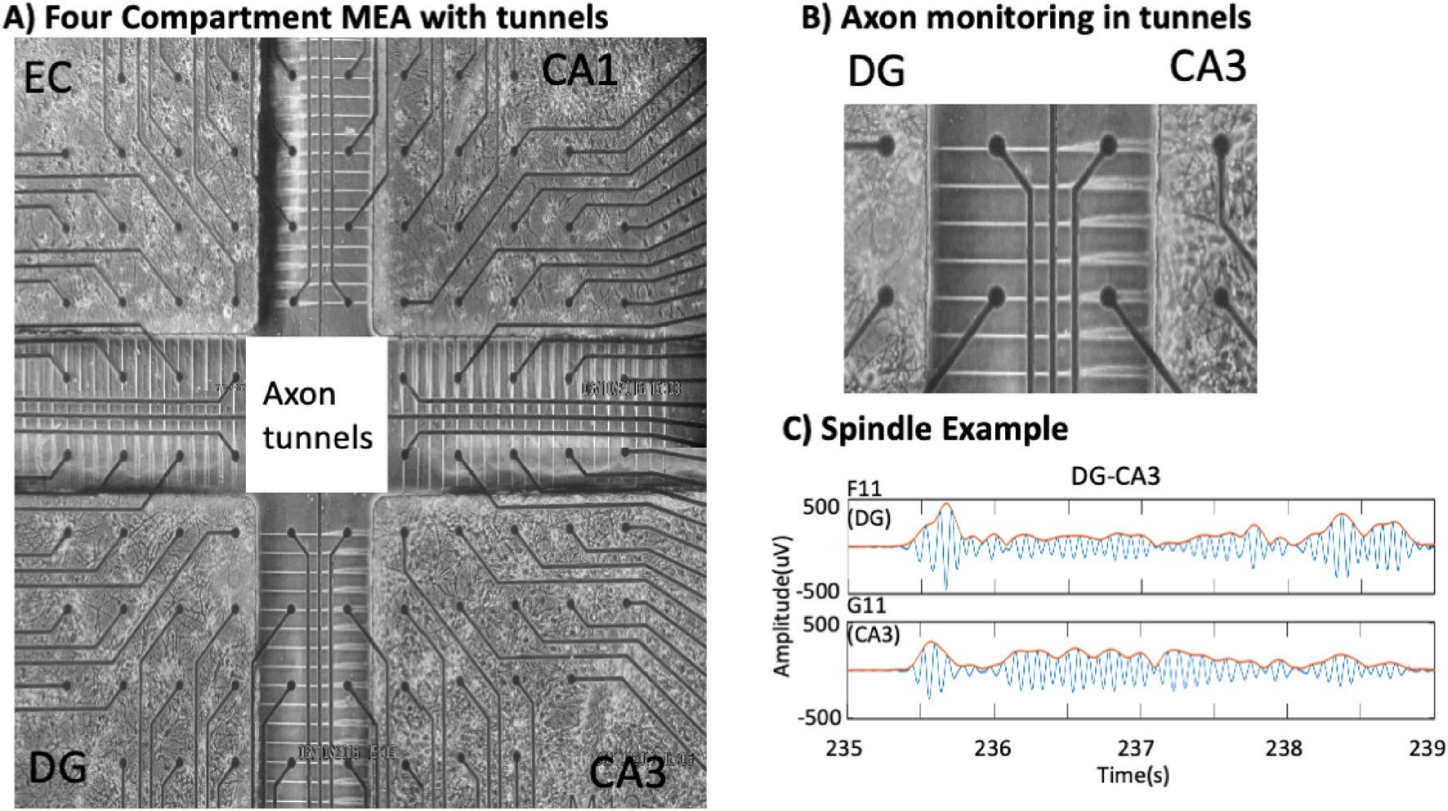
(**A**) Four-compartment device for measuring spindle oscillations in axons between separate subregions of neurons from the indicated subregions of the hippocampal formation on top of an electrode array. Subregions were connected by microfluidic tunnels for axonal communication. Electrodes were the 30 μm diameter dark circles, spaced 200 μm apart. Region in the white dashed box was enlarged to show in (**B**) two electrodes in each tunnel allowed monitoring direction of axonal conduction and detection of (**C**) spindle oscillations filtered for frequencies of 10–16 Hz in an axon passing from the CA3 back to the DG.

In a unique approach, we cultured hippocampal neurons from each subregion of the rat hippocampal formation over an electrode array to measure spindle oscillations^15^. Silicone rubber (polydimethylsiloxane, PDMS) compartments separated four individual subregions of the hippocampal formation: neurons from the EC, dentate gyrus (DG), CA3 and CA1. The PDMS device was microfabricated with narrow microfluidic tunnels (10 µm wide × 400 µm long × 3 µm high) that excluded somata and dendrites but allowed axons to communicate between the subregions in a closed loop architecture (Fig. 1A, B). Two electrodes separated by 200 µm in a few of the tunnels allowed measurement of the direction of propagation of action potentials in single axons. To our surprise, we detected 0.5–2 Hz slow waves and 10–16 Hz spindle oscillations in axons. The small cross section of the tunnels and their high resistance indicated spindle origins as changes in the axon membrane potential, independent of action potentials, independent of thalamic inputs and independent of volume conduction suggesting a new mechanism for generation and transmission of spindle oscillations.

## Results

We reverse engineered the rat hippocampus in order to enable precision access to differences in information processing in the four subregions of the input signal from the EC and downstream processing in the DG, CA3, CA1 (Fig. 1). The subregions were interconnected by microfluidic tunnels that isolated individual axons^15,16^ including a return route to the EC. By filtering the raw data at 10–16 Hz, our 30-µm extracellular electrodes detected oscillations (Fig. 1C) similar to sleep spindles seen in EEG and rat^10,17^ and human intracranial recordings^6,18^. Inspection of videos of these signals in CA3 that feed forward to CA1 (Supplementary Fig. S1A) and back to DG (Supplementary Fig. S1B) reveal numerous spindle frequency oscillations associated with large slow waves > 300 ms in length. Many short spindle-band events are seen that were below our length threshold of 300 ms and not evaluated as spindles.

**Figure 2:**
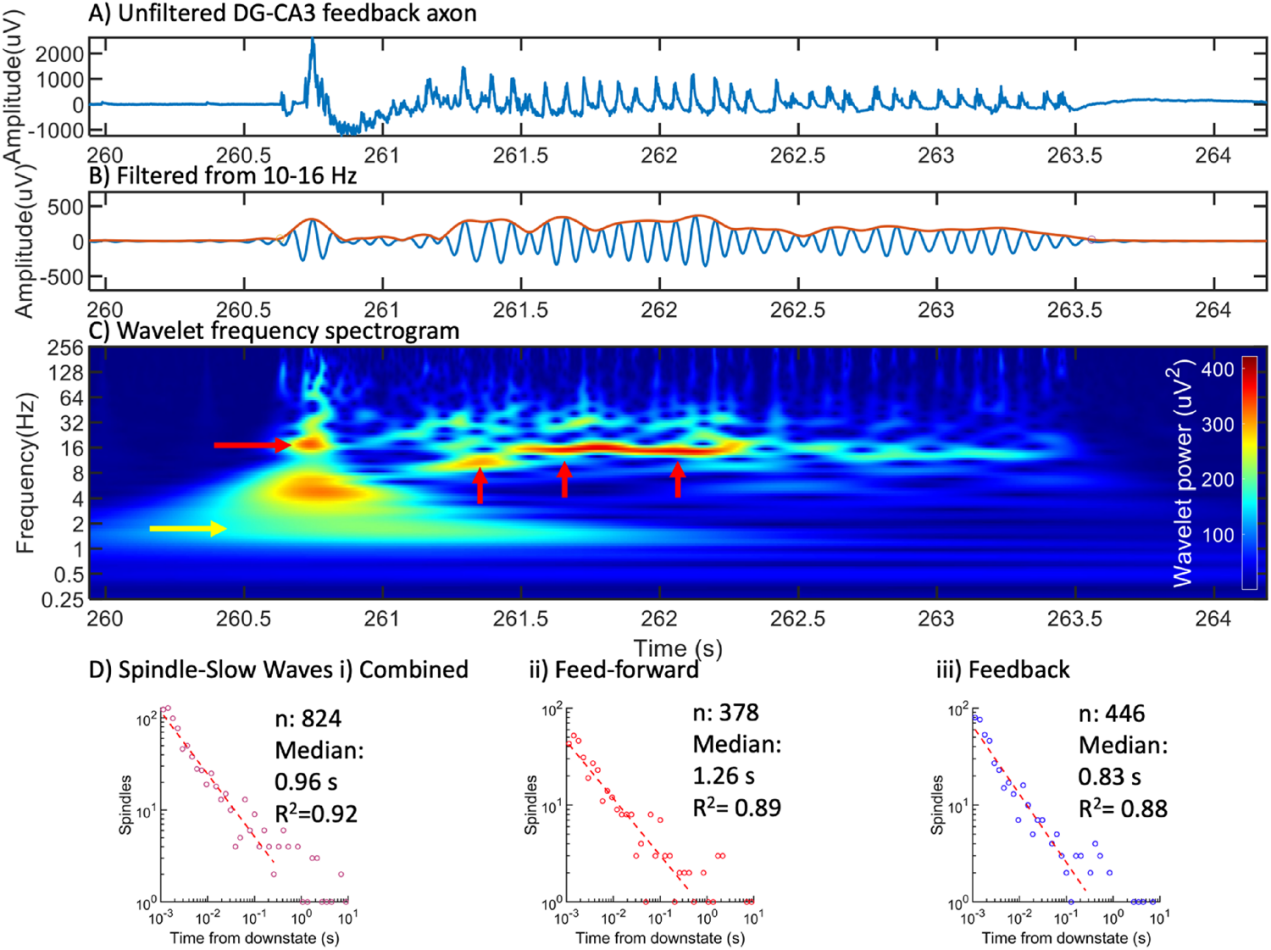
Spindle frequencies embedded in the spike train. (**A**) Raw data of single axon from CA3 feedback to DG (F12-G12) channels, array ECDGCA3CA1 19908 160518 160610 d22). (**B**) Same data filtered from 10 to 16 Hz and a Hilbert-transformed envelope to reveal several spindle events. (**C**) Continuous wavelet transform to show wave power and relative timing at different frequencies. Red arrows point to spindle events. Yellow arrow points to slow wave near 1.5 Hz. (**D**) Log–log distribution of spindle events relative to slow waves for all subregions, (i) feed forward and feedback in all subregions combined, (ii) feed forward spindles, (iii) feedback spindles. Best fit linear models are overlayed.

A typical workflow is shown in Fig. 2. The signal in Fig. 2A shows a 4 s portion of raw data rich in oscillations in these narrow, high resistance tunnels of 3 × 10 × 400 μm. Embedded in this signal were low amplitude oscillations evident by filtering at 10–16 Hz (Fig. 2B). The slow onset, peak and decline over 3 s were characteristic of spindle waves^1^. An envelope was determined with a start and stop time based on the Hilbert transform and thresholding. The relationship of these spindles to other oscillation frequencies is shown in Fig. 2C. The power distributions of spindle frequencies for all subregions peaked at the low end of the 10–16 Hz range, around 10 Hz (Supplementary Fig. S2). Other regions of activity showed some overlap with higher frequency gamma oscillations, most of which were coincident with spikes and not spindles. This spindle begins at 260.7 s with a sharp wave ripple (10–30 Hz), theta waves (4–8 Hz) and slow wave oscillations (0.5–4 Hz). To determine if spindles were evoked by slow waves, we examined the histogram of spindle counts with respect to the onset of each slow wave in single axons (Fig. 2D). As in^19^, spindles often occurred within 1.5 s of the onset of a slow wave. The histogram was log–log distributed, suggesting dependence on a number of non-linear events. While all spindle events were log–log distributed (Fig. 2Di), separation into feed forward (Fig. 2Dii) and feedback events (Fig. 2Diii) demonstrated good fits over 2.5 orders of magnitude. Feedback events occurred at 30% shorter times (median 0.83 s) than feed forward (1.26 s). Supplementary Fig. S3 shows the distributions within component subregions that contributed to the summary in Fig. 2D. The shortest median of 0.65 s was seen in EC-CA1 feedback spindles, suggesting a tighter coupling there for slow wave contributions to spindle generation.

### Slow wave time delays compared to spike timing

**Figure 3:**
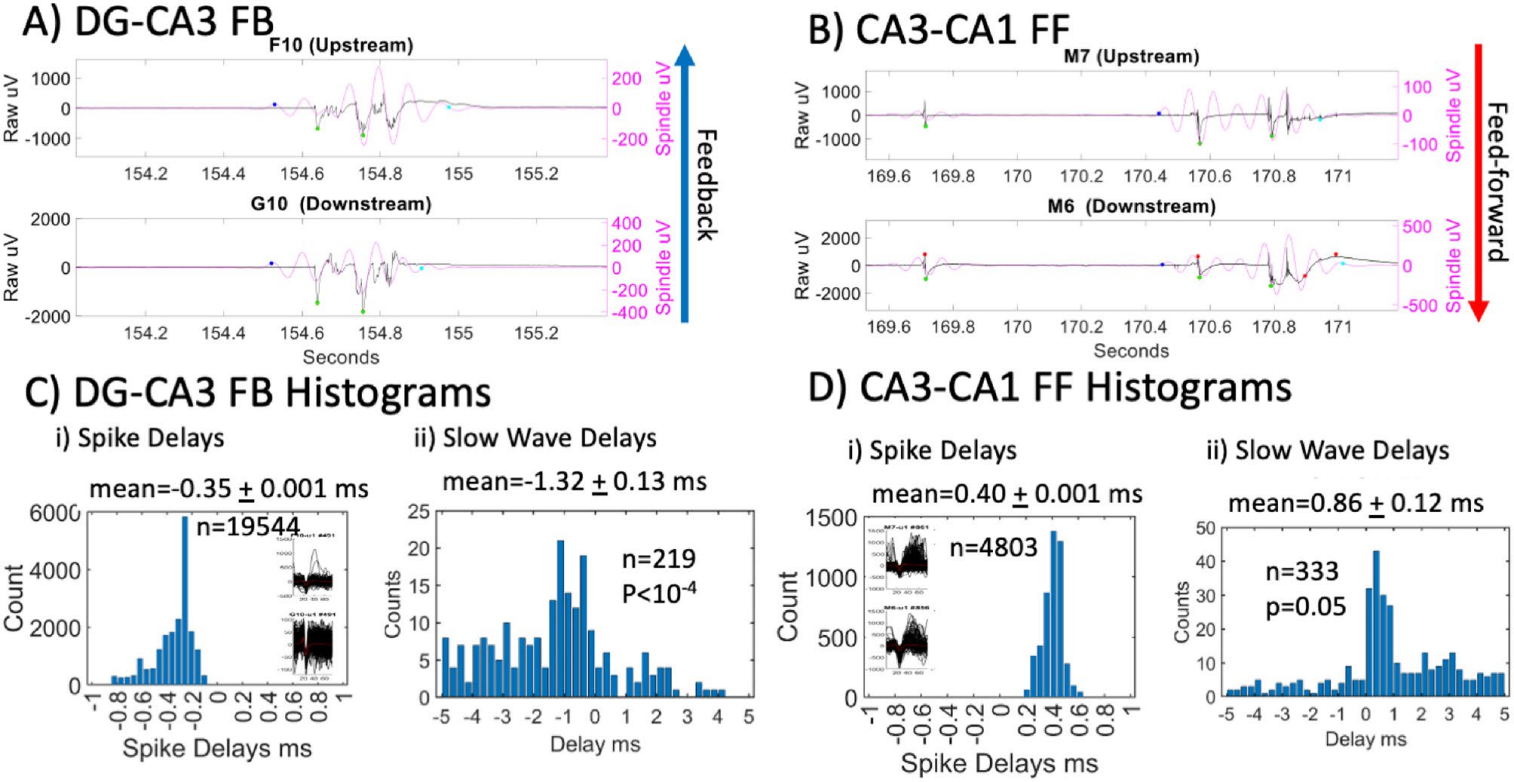
Comparison of slow wave-spindle timing and spike timing of single axons traversing two electrodes in a tunnel. (**A**) and (**B**) raw data with overlay of spindle oscillation from upstream and downstream tunnel electrodes for a signal determined by spike timing to be an (**A**) feedback axon from CA3 to DG or (**B**) feedforward axon from CA3 to CA1. Blue dots in A and B mark the start of the spindle and cyan dots the end of the spindle. Green dots are points that match the slow wave minimum in both channels. Red dots are unmatched minima. (**C**) and (**D**) Corresponding histograms of (**C**i) mean spike delays for feedback axons between two electrodes in tunnels between DG and CA3. Insets show Wave_clus waveforms for all n spike shapes, centered at sample number 200 (25 kHz) on x-axis. (**C**ii) mean slow wave delays on the same axons. 15 single axon spike timing delays from 9 arrays. *P* value is t-test of slow wave delay significantly longer than spike delay. (**D**i) mean spike delays for feed-forward axons between two electrodes in tunnels between CA3 and CA1, (**D**ii) mean slow wave delays on the same axons. 5 single axon spike timing delays from 9 arrays. P value is t-test of slow wave delay significantly longer than spike delay Spike bins are 0.16 ms and spindle bins are 0.25 ms.

If spike timing drives the spindle oscillations, then the timing of axon spikes and slow waves over the two tunnel electrodes will be the same. Supplementary Fig. S1 shows spindles mostly with spikes, but they may or may not be causal for slow oscillations. Analysis of timing delays of the peak in spindles between the tunnel electrodes proved exceedingly unreliable with standard deviations of delay times exceeding the means. This was probably due to how the spindle filter creates a sine wave based on the variable positive vs. negative amplitude variations in the first slow wave, it’s voltage asymmetry and amplitude relative to the next slow wave. Figure 3 shows examples of slow waves with embedded spindles in feedback axons from CA3 to DG (Fig. 3A) and feed-forward axons from CA3 to CA1(Fig. 3B). These more precise troughs were used for calculating the delay time from one electrode to the next one in the tunnel 0.2 mm away are marked with green dots. The corresponding spike time delays and slow wave time delays are shown in Fig. 3C, D. Similar to previous analyses^15,16^, the Fig. 3C axon spike delays of 0.35 ms correspond to a CA3-DG feedback conduction velocity of 0.57 m/s. For the same axons, the slow wave conduction delay was 1.32 ms or 0.15 m/s, 74% slower. The difference was significant by t-test (*p* < 10^−4^). In Fig. 3D, CA3-CA1 feed forward spike delays of 0.4 ms equal a mean spike conduction velocity of 0.5 m/s, while slow wave delay times averaged 0.86 ms or 0.23 m/s. The 54% slower velocity of the CA3-CA1 slow wave compared to spike velocities was also significant at *p* = 0.05. In summary, spindle oscillations appear independent of spikes since their conduction velocities are significantly slower than spikes.

### Spindle dynamics

**Figure 4:**
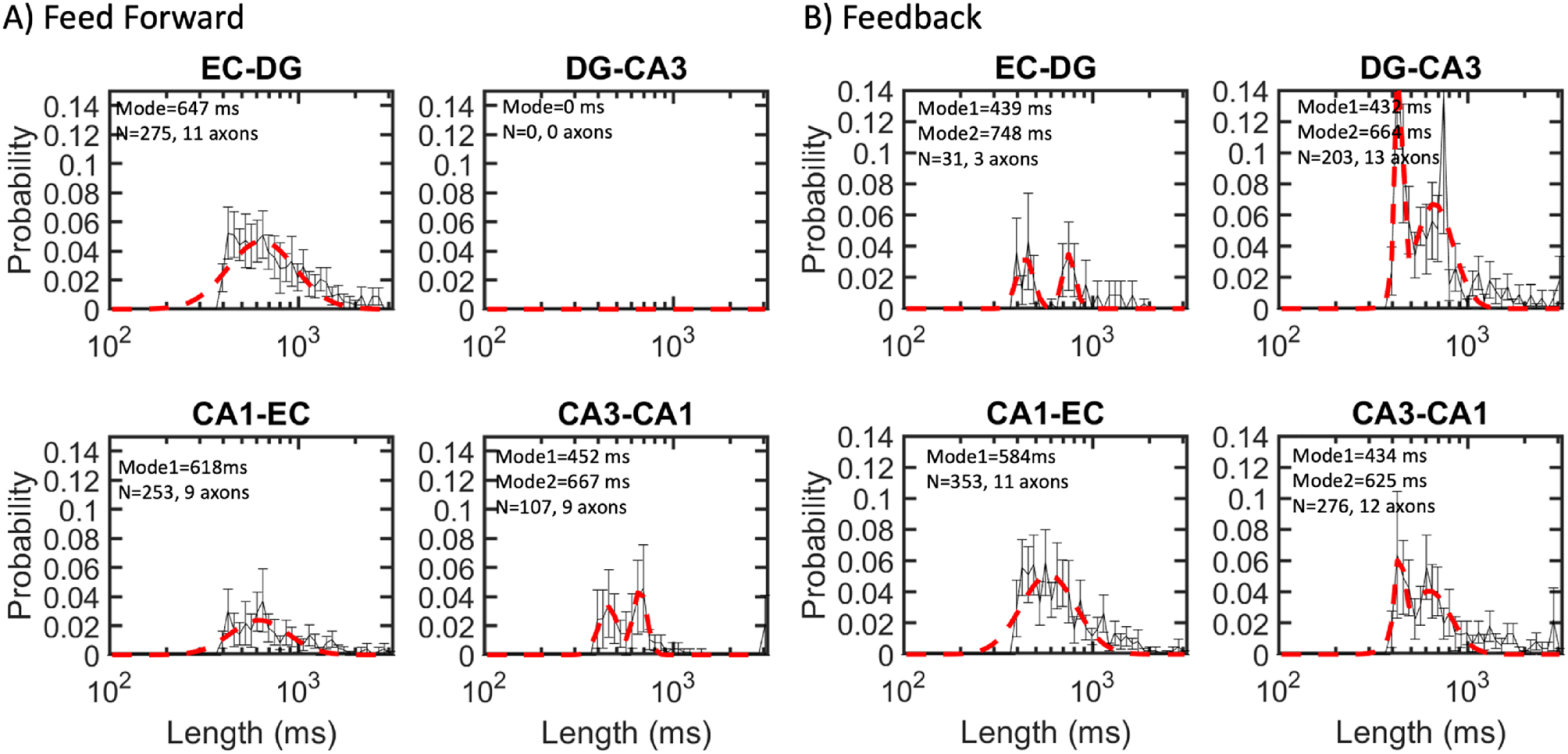
Spindle length modes ranged from 430 to 750 ms in semi-log distributions of all spindles from nine arrays combined. (**A**) Length of spindles of feed forward axons normalized across feed direction for EC-DG, DG-CA3, CA3-CA1, and CA1-EC. (**B**) Spindle length for feedback channels. Data from DG-CA3 feed forward was ignored because of low event counts. Data was fit into 50 log bins. N is the number of spindles on a given number of axons.

We characterized the duration, amplitude, and spacing of these spindle waves in each of the four sets of axon tunnels that connected adjacent subregions. The length of spindles followed a semi-log Gaussian distribution with modes ranging from 430 to 750 ms (4–8 cycles), although long tails in the distributions showed some spindles with durations as long as 2 s (Fig. 4). The feed forward CA3-CA1 distribution and three of the four feedback spindle lengths were fit with two peaks. Four of these longer dual peaks and the three single peaks averaged 650 +/− 21 ms (S.E.). Shorter peaks averaged 439 + / − 5 ms. These durations were similar to the 0.4 s prevalence seen in rat hippocampus in vivo^10^. Feed forward axon spindle lengths showed significant subregional differences by ANOVA (F(634,2) = 4.2, *p* = 0.01 as were feedback axons F(862,3) = 17, *p* < 0.0001. The number of spindles in feed forward EC-DG axons (275) was 9× greater than the feedback EC-DG axons (31). In all the other axons, feedback spindles (832) were 230% more prevalent than feedforward (360). These similar spindle lengths suggested a similar mechanism of generation of the rise and fall in amplitude of each axon spindle, predominantly in feedback axons.

**Figure 5:**
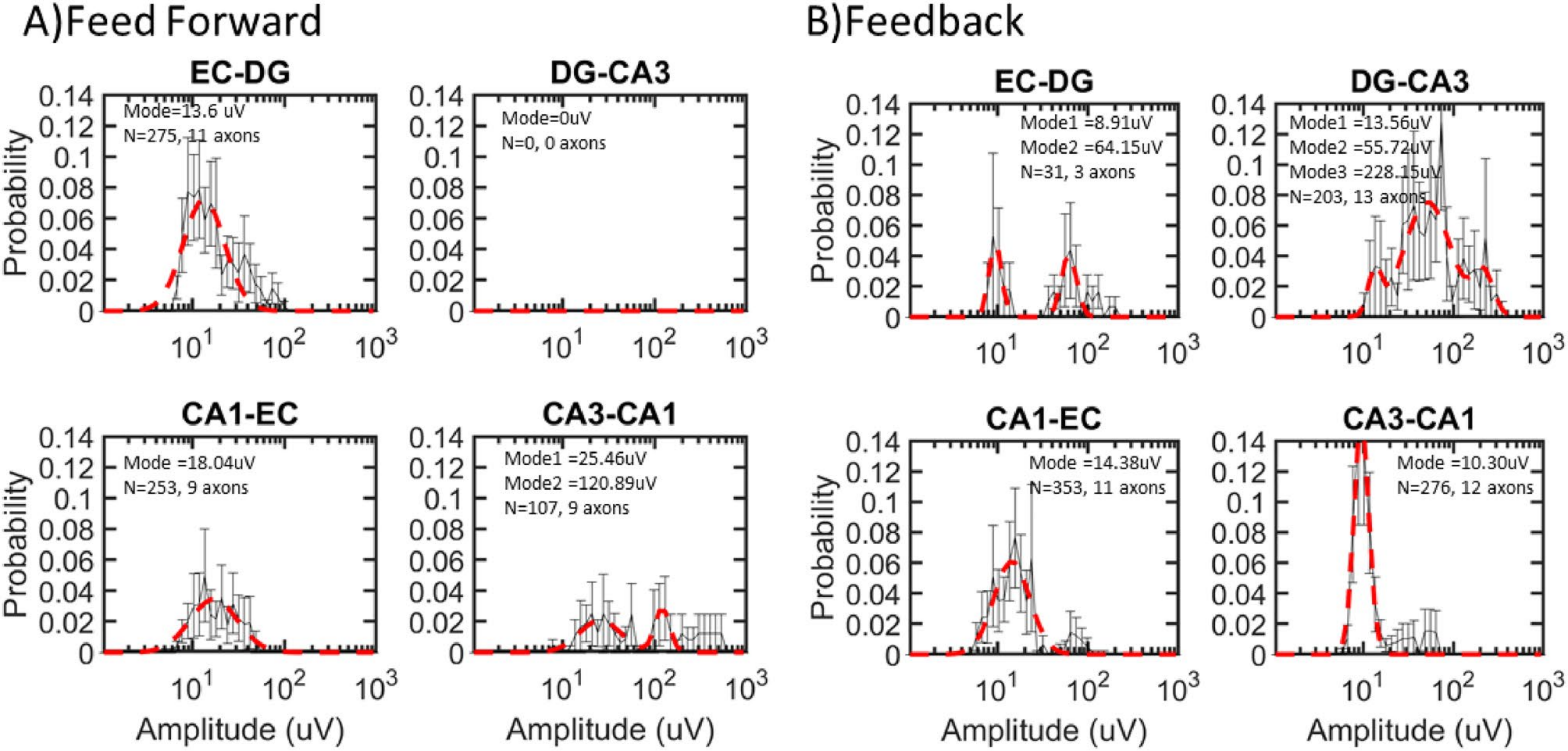
Largest amplitude spindles in DG-CA3 feedback (CA3-DG) and CA3-CA1 feed forward from nine arrays combined. (**A**) Amplitude of spindles of feed forward axons normalized across direction for EC-DG, DG-CA3, CA3-CA1, and CA1-EC. (**B**) Spindle amplitudes for feedback axons. Data was fit into 100 log bins. Data from both directions from each subregion were combined to normalize to a total probability of 1. Data was fit into 50 log bins.

We propose that the highest amplitude spindles might be the spindle source within the hippocampal network. Figure 5 shows that amplitudes were well fit by semi-log Gaussian distributions. The subregional axons with the highest peak amplitudes were CA3-CA1 feed forward (121 µV) and DG-CA3 feedback (228 µV) with long tails extending to even higher amplitudes. Since these high amplitude spindles share the common CA3 subregion, this suggested that the CA3 subregion was the source of high amplitude spindles within axons in both directions within these networks. Other feed forward modes were only 14 µV for EC-DG and 18 µV for CA1-EC. CA3-CA1 feed forward axonal spindle amplitudes were different from the others by ANOVA F(634,2) = 85, *p* < 0.001, and all feedback axon distributions were different F(862,3) = 65, *p* < 0.0001.

**Figure 6:**
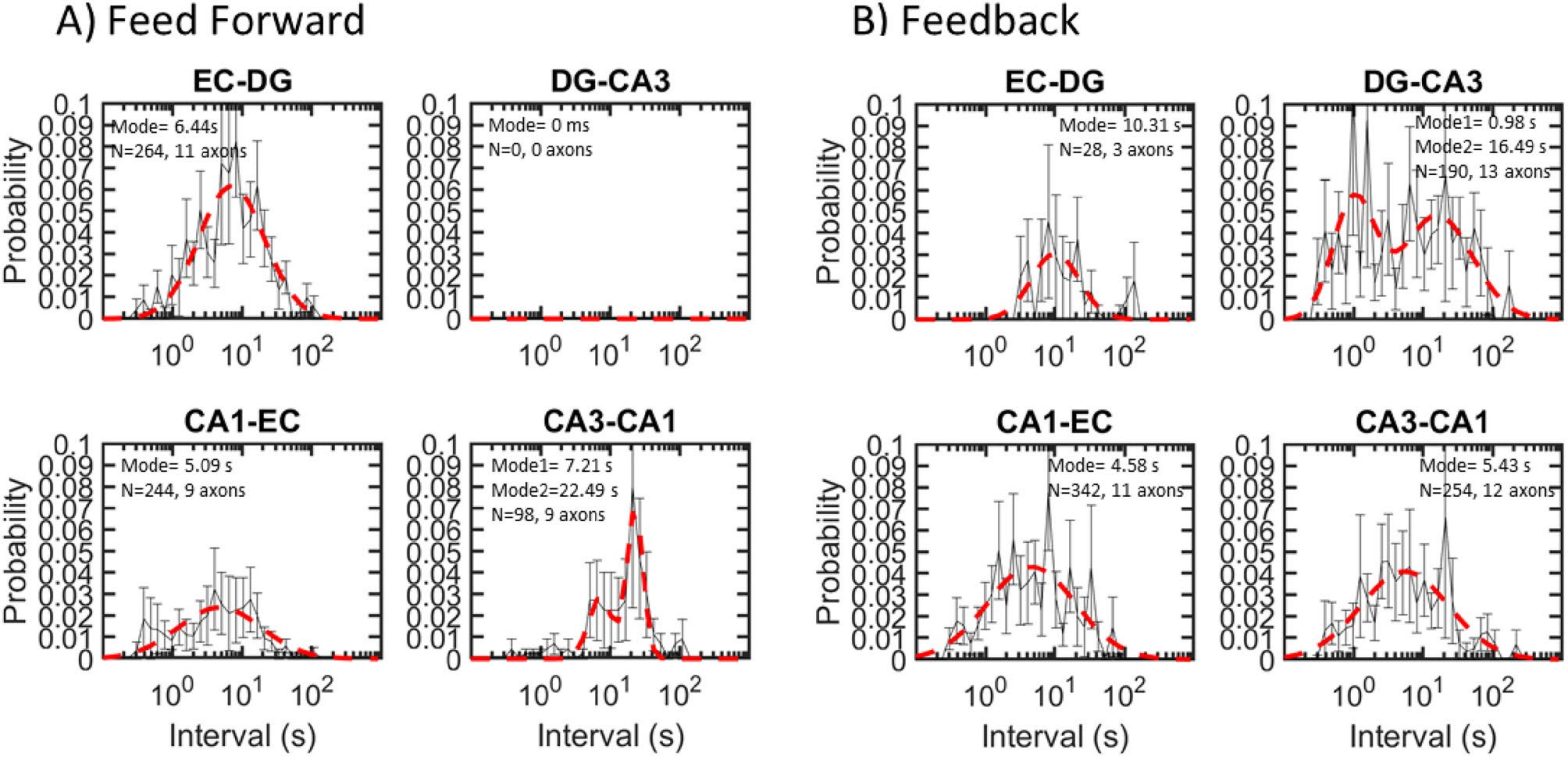
Shortest 1 s inter-spindle intervals were in feedback DG-CA3 axons. (**A**) Inter-spindle intervals of feed forward axons from nine arrays combined and normalized across feed direction. (**B**) Inter-spindle interval for feedback axons. Data from DG-CA3 feed forward axons were ignored because of low event counts. Data was fit into 50 log bins.

We measured the inverse of the rate of spindle generation as the inter-spindle interval (Fig. 6). Interval peak modes in the semi-log distributions of 1 s were shortest for feedback axons CA3 to DG. Other subregions ranged from 6 to 10 s. These times were similar to sleep spindle distributions from Fz and Cz scalp EEG electrodes^20^. Axonal inter-spindle-intervals of the feed forward subregions were different from the others by ANOVA F(605,2) = 19 (*p* = 0.01) as were feedback axons F(823,3) = 11 (*p* < 0.001). The fastest 1 s intervals in CA3-DG feedback axons equated to 1 Hz, which was the frequency of slow oscillations seen by others in the rat CA1^21^.

### Correlations of spindle phase with spike times in axons

**Figure 7:**
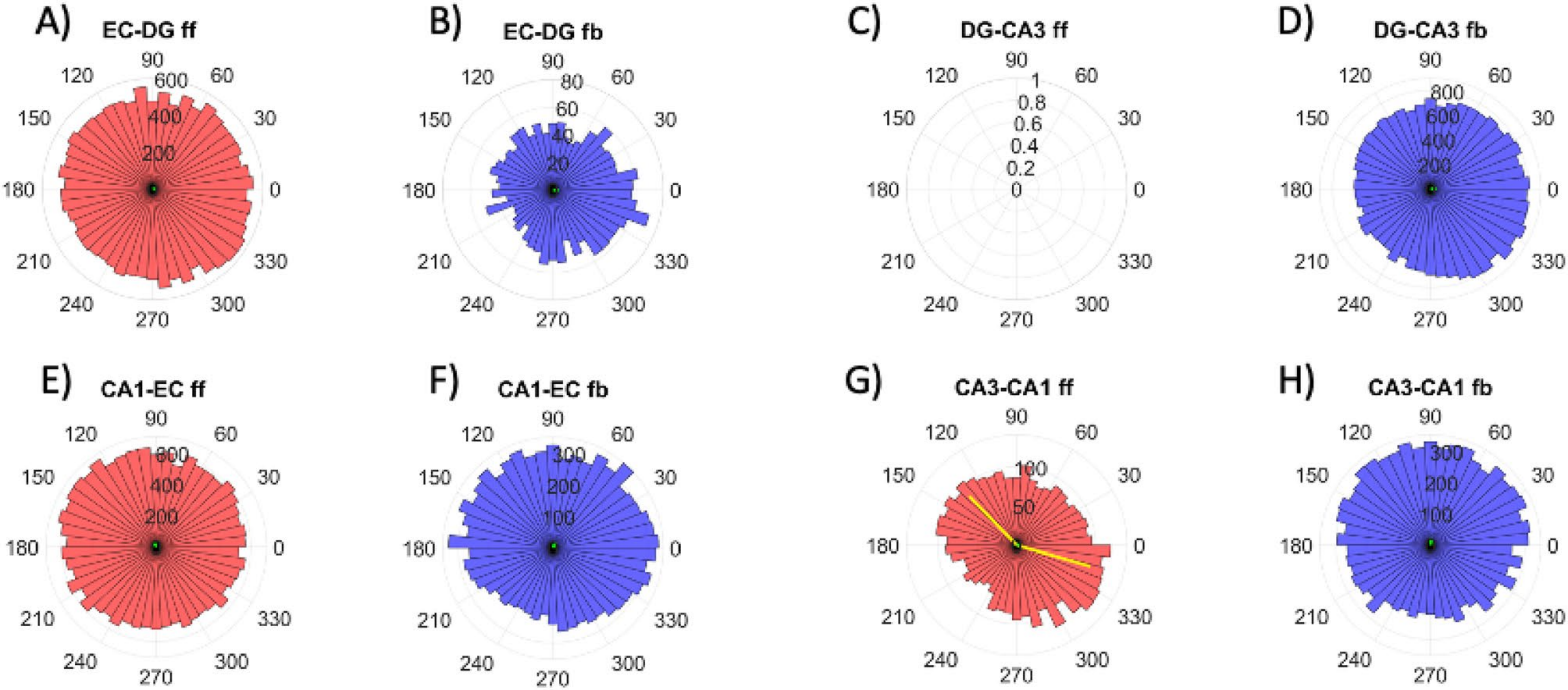
No correlation of spindle phase with spike times. Polar histograms to correlate spikes with the phase of the spindles from nine arrays. (**A**–**H**). Each axon compartment and direction is shown in a separate graph. Red are feed forward axons (ff) and blue are feedback (fb). Polar plots cover 360 degrees of each complete wave cycle within a spindle, starting at the zero crossing to a peak at 90 and trough at 270. Lengths of normalized vector sums of directions were close to zero in all cases except G. Only (**G**) CA3-CA1 feed forward axons show spike times bimodally enriched at the descent from the 90 degree peak at 130 degrees and the rise from the 270 degree trough at 345 degrees, but the effect size was only 0.1. The largest effect size was in B, still small at 0.27.

To determine if the phase of the wave oscillations in the spindles correlated with spiking, polar plots were used to reveal phase enrichment, either in the feed forward or feedback directions for each subregion of axonal connections. Looking at times when there was a spindle in each subregion, Fig. 7 shows that only in the CA3-CA1 feed forward direction (Fig. 7G) was there a correlation of the spikes with the 130 and 345 degree phases of the spindle cycles at the descent from the 90 degree peak and at the rise from the 270 degree trough (*p* = 0.001) but the effect size was only 0.1. Spikes were only randomly correlated with spindle phase in all the other directions and subregions (effect sizes ranged from 0.02 to 0.3, virtually zero). Therefore, axonal spikes were not phase locked with spindles.

### Correlations of subregion neuron spindles with axon spindles

**Figure 8:**
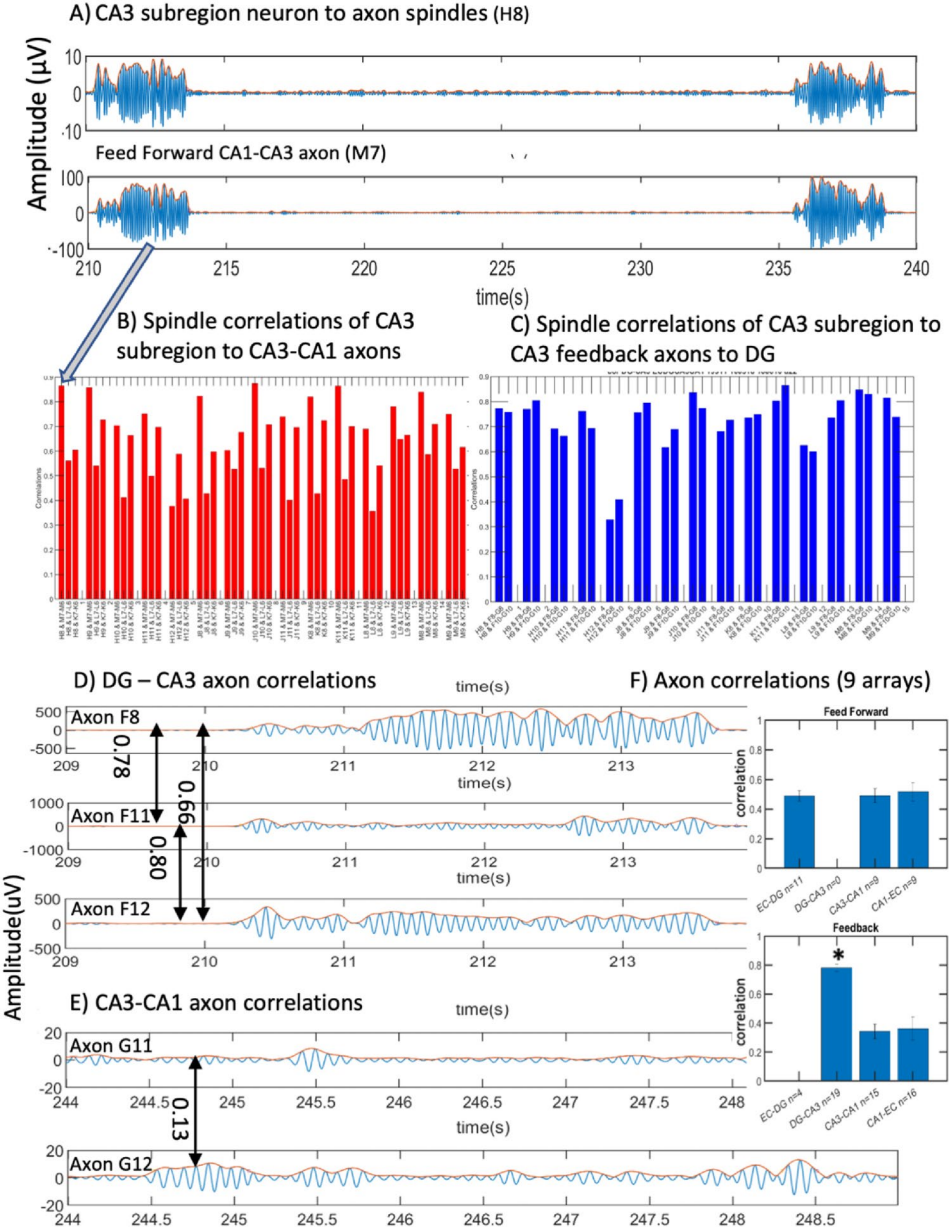
Spindle envelope correlations to find direct connections between CA3 subregion neurons and their axons. (**A**) Comparison of CA3 subregion spindles (H8) to feedback CA1-CA3 axon spindles (M7) with a time lag of 7 ms over an undetermined path > 0.8 mm. (**B**) Correlations between 17 CA3 subregion neurons and spindles from one CA3-CA1 axon. Only one axon in this array showed spindles. (**C**) Pearson correlations between CA3 subregion electrodes with CA3-DG feedback axons. All permutations of subregion electrode spindles with axon spindles are shown for one array in groups of the three active axons with each of 17 subregion neurons. (**D**) Moderately strong axon to axon correlations for spindles in three axons communicating from CA3 to DG (feedback). (**E**) Example of poor correlation for spindles on two axons communicating from CA3 to CA1 (feedback). (**F**) CA3-DG feedback axon spindle correlations strongest among other subregions over 9 arrays. *ANOVA F(25,2) = 27, *p* = 10^−6^. Numbers of EC-DG feedback axons were insufficient to compare two axons in the same array.

Our device contained separate compartments for principal neurons of each subregion and their axonal afferents to the adjacent subregion. If the spindle signal arose from volume conduction, all subregion signals would be highly correlated. If the spindle voltages arose from membrane potentials, then only neurons in a cell assembly and their afferent axons would be correlated with direct outputs approaching a correlation of 1. Figure 8A shows the correlation of two axonal spindle oscillations from CA1 back toward CA3 into a spindle in the CA3 subregion neurons. The delay time of 7 ms and the 0.82 mm minimum distance resulted in a minimum spindle velocity of 0.1 m/s. The location of each electrode is provided in Supplementary Fig. S4. For this example, Fig. 8B shows a high correlation, r > 0.9, for spindles on this one axon onto 12 of 16 spindles on target neurons in the CA3 subregion. This suggested prodigious connectivity of this feedback neuron onto the subregion principal neurons. Connection of this axon to the other 4 target neurons was weaker and ranged from 0.65 to 0.85, which are still considered strong correlations. For two CA3-DG feedback axons onto their target neurons in CA3 (Fig. 8C), the variations in correlation of spindles were greater ranging from 0.3 to 0.85. Also, the paired bars show that spindles from two different axons targeting the same DG neuron were correlated differently. Another point on correlations, we detected 20 axons in total with spindle activities but no active spiking activity. Two of them are located in EC-DG, two in DG-CA3, six in CA3-CA1, and ten are in CA1-EC. Since not all correlations were equal, we concluded that spindle conduction proceeded by propagating oscillations in membrane potential on axons, independent of action potentials, rather than by volume conduction.

We explicitly examined the spindle correlations between the axons in the DG-CA3 region (Fig. 8D). These examples show some of the higher correlations for DG-CA3 axons. Figure 8E shows examples of the lower correlations for CA3-CA1 axons. Averaging nine arrays, Fig. 8F shows that feed forward axon spindles were largely independent with r = 0.5 (F(23,2) = 0.07, *p* = 0.9). Only CA3 feedback to DG axons spindles were significantly more correlated (r = 0.8) than the spindles on axons from other regions (r = 0.3, by ANOVA F(25,2) = 27, *p* = 10^−6^). This feedback synchrony from CA3 to DG might contribute to limiting the detonation, winner-take-all firing in DG^22^. Overall, the variability of spindle correlations between axons and target or source neurons argued against volume conduction of spindle oscillations and a more specific role of axonal spindle oscillations in regulating synaptic plasticity.

Are slow waves and associated spindles the result of passive conduction from somal current sources? If slow wave conduction is passive, then the magnitude of the slow wave and embedded spindle oscillations in an axon will be smaller with distance over the 0.2 mm spacing of the two electrodes in the tunnel. Since amplitudes were strongest in feed-forward and feedback axons connected to the CA3 neurons, we examined these axons for relative magnitudes of their slow waves. CA3-CA1 feed-forward axons closest to the CA3 subregion had a mean amplitude of − 650 μV while the further electrodes were − 679 μV. A paired t-test of slow wave amplitudes by axon was *p* = 0.68 (n = 5 axons) for a decline in amplitude with distance. Similar analyses of the CA3 to DG feedback axons produced mean amplitudes of − 1420 μV closer to CA3 and − 832 μV away; however, the paired t-test was not significant at *p* = 0.08 (n = 9 axons). We conclude that factors of axon coupling to the electrode were more important to slow wave and spike amplitudes and that evidence was not found for passive cable conduction of slow waves and embedded spindles.

## Discussion

Two novel findings are reported for 10–16 Hz spindle oscillations in hippocampal axons. (1) Spindle oscillations were transmitted in single axons with important implications for their origins, field potentials, volume conduction and their relationships to spikes. (2) Spindle oscillations emerged from a reverse engineered hippocampal network, independent of the thalamus with possible implications for function in cognitive consolidation. Our in vitro system has obvious limitations in that recordings could not be correlated to sleep stage or memory consolidation. Features in common with in vivo spindles included prominent waxing and waning oscillations of 10–16 Hz that were detected in the single axons passing through the 3 µm high tunnels with semi-log distributions of durations centered around 0.5 s but with long tails up to several seconds both in feed forward and feedback directions. Their semi-log distributed amplitudes exceeding 100 µV in CA3 axons feeding back to the DG, and CA3-CA1 feed forward axons, suggesting an origin in CA3 neurons. CA3 back to DG and EC to CA1 feedback axons were of the shortest mode of inter-spindle intervals at 4–5 s (0.2 Hz) but ranged down to 0.5 s (2 Hz). These intervals are consistent with modulation by slow waves^19,23^. Other features could be compared to the in vivo recordings from rats and humans. For rats with implanted electrodes into the hippocampus, Sullivan et al.^10^ found evidence for sleep spindles throughout the hippocampus with most prominent source and sinks in the CA1 stratum lacunosum moleculare. This region could be the target of the feed forward axons that we observed from CA3 to CA1, but also evoked the strong modulatory feedback responses we see. In vivo oscillations in single axons are technically impossible to detect. Sullivan also observed virtually all rat spindle durations between 0.4 and 0.8 s, similar to our observations, and similar to the spindle durations reported in human intracranial scalp EEG recordings of 0.5–1 s (mean 0.68 s)^18^. These human recordings also showed a spindle frequency of 0.85/min from the temporal lobe, far slower than our hippocampal axon recordings at 12/min, but similar amplitudes of 120 µV. Given the dynamic similarities of our hippocampal spindles to those in rats and humans, we now turn to the uniqueness of our reconstructed model hippocampus.

### Spindles in single axons

These were the first recordings of spindle frequency oscillations in single axons. We know they were single axons because of the uniform waveforms detected at a predominant delay time or conduction velocity over the 0.2 mm inter-electrode distance in the 3 µm high tunnels^15,16^. As such, we were able to monitor with small 30 µm diameter extracellular electrodes sizable 100 µV oscillations produced by local membrane currents. The narrow 3 × 5 µm cross section of the tunnels produced a high impedance pathway, shielding out any volume conducted field potentials. Field potentials are often detected in brain tissue with 0.1–1 mm or larger electrodes, precluding assignment to individual neurons and certainly not axons. Most of the axons in vivo in the hippocampus can only be recorded from a bundle, such as the Schaefer collaterals from the CA3 to the CA1. This anatomy makes identification of the extent of communicating synchrony unavailable to dissecting the specificity of information communicated between subregions of the hippocampus as we have done here and previously reported^15^. We did measure a moderate correlation of feed forward spindle oscillations between subregions, and a more substantial r of 0.7 for the feedback CA3-CA1 axons. However, to reach the scalp, numerous axon tracts, most likely in the proximal cortex, must be oscillating together at spindle frequencies during non-REM sleep. These oscillations are usually recorded as local field potentials and assumed to be volume conduction. However, a field potential must arise from aligned dipole potentials, which in turn must arise from coincident currents. Since the field potential from spiking of a single axon attenuates too rapidly to reach more than even 100 µm beyond its source^24,25^, we concluded that better methods were needed, which were what we have provided in this report. Ion channels in axons are not usually considered. Instead, currents arising from coincident EPSC’s are often ascribed as sources for oscillations. Therefore, our novel measurements of oscillatory potentials in axons suggested oscillations in the axonal membrane potential. Further, their independence from action potentials suggested a second source of voltage-dependent ion channels, different from the classic sodium and potassium channels with sub-millisecond kinetics. Steriade et al.^26^ originally described 0.5–4 Hz slow waves as oscillations in membrane potential by in vivo sharp electrode recordings from thalamic somata in the anaesthetized cat. Sullivan et al.^10^ also concluded that the in vivo rat hippocampal spindles were not volume conducted, and proposed a possible origin in spikes that were phase locked to the spindles. However, their in vivo recording of rat hippocampus exhibited no correlation of CA1 spindle phase with spiking in principal cells or interneurons, similar to the absence of correlation that we observed and their lack of co-modulation in human intracranial depth electrodes^6^. Active ion channels in axons separate from those that propagate spikes may be similar to the active calcium channels in dendrites^27^. Our failure to find consistent loss of slow wave amplitude with distance along the axon suggests that slow wave-spindle wave oscillations are not passive electrotonic conductances originating in their somata or dendrites. Identification of the spindle channels in axons awaits pharmacological inhibition, but could have implications for sleep pathologies, and treatments.

### Spindles in the hippocampus, independent of the thalamus

Steriade et al.^8^ found spindles in cortical slices of cat brains that included the thalamus. Surgical isolation of the reticular thalamus away from the cortex preserved these oscillations allowing the authors to conclude a thalamic origin for spindles. However, they did not examine the hippocampus in their preparation where others have observed spindle oscillations in the in vivo rat^10^ and human brain^6^. Andrade et al.^11^ found human connectivity of the hippocampal formation to the prefrontal cortex during spindle oscillations in S2 by fMRI, but stronger sleep spindles in the hippocampal formation during slow wave sleep without cortical connectivity. This suggested at least periods of segregated spindle-related processing within the hippocampus. Similarly, depth electrode studies in human seizure patents suggested a thalamic origin of sleep spindles^6^, however 48% of all spindles they measured were in isolated brain regions, independent of thalamus. In the hippocampus, they occurred at 1.1 spindles/min., less frequent than scalp EEG at 1.9 spindles/min.

By the same logic, we isolated neurons from the rat hippocampus, disconnected from the thalamus and found spindles of similar oscillation frequency (10–16 Hz), oscillation duration (modes of 430–750 ms, but extending longer) and faster frequency of occurrence (10 spindles/min). Since these hippocampal spindles occurred without a thalamus, we concluded that they were generated locally, consistent with Andrillon’s findings^6^. Although spindles were detected in all feed forward and feedback directions except for DG-CA3 feed forward axons, the highest amplitude spindles occurred in axons connected to the CA3 subregion (CA3-CA1 feed forward and CA3 back to DG). This subregion was not previously studied in vivo, since CA1 was preferred for its higher spike rates^10^. Spindles in other regions of different amplitudes, different inter-spindle timing, and variations in correlations between axons suggested independent origins in separate subregions. These differences indicated discrete axonal transmission from oscillatory changes in membrane potential and argued against volume conduction. Their independence from spikes and 54–74% slower transmission velocities also suggested a mechanism of voltage-sensitive membrane ion channels independent from those responsible for action potentials. The amplitude and frequency of this emergent rhythmic activity depend on intrinsic cellular properties and the connectivity and strength of both excitatory and inhibitory synapses^28^. There is no doubt that a field potential arose from every membrane current, but the substantial potentials observed at the scalp and from large intracranial electrodes and the fall off of potentials at 1/r^29^ requires the additive potentials of hundreds of synchronized axonal oscillations in fiber tracts in gyri perpendicular to the surface. In thick thalamo-cortical slices from postnatal mice, spindle waves propagated at 0.11 mm/s in the greater horizonal dimension and 2.4 mm/s vertically^30^. Here we measured the individual axonal source of these oscillations at 0.15–0.23 m/s, far slower than spikes at 0.5–0.6 m/s^15,16^.

In the broader context of sleep spindles and memory consolidation, Peyrache et al.^31^ found functional deafferentation of the cortex from its hippocampal inputs, a kind of gating presumably mediated by the strong recruitment of inhibitory interneurons. In contrast to cognition, consolidation of motor memories or tasks might require transient connection to thalamus during sleep^32^. In either motor or cognitive consolidation, the combined slow wave and spindle oscillations could contribute to credit assignment within the network^33^. The hippocampus might keep memory in context for several days before passing events on to the cortex for thalamic activation by spindles^34^. We recognize direct anatomical reciprocal connections of the thalamus and the hippocampus^35^ that could transmit or receive spindles at specific times, but here we have demonstrated that the hippocampus by itself was able to generate spindle oscillations, independent of the thalamus. When the phase of these spindle-associated slow waves was augmented by deep brain stimulation in human prefrontal cortex, recognition memory was enhanced the next day, demonstrating their role in consolidation^5^.

## Conclusions

We found 10–16 Hz spindle oscillations in single rat hippocampal axons in both feed forward and feedback directions among the four subregions of a reconstituted hippocampal formation. The spindle oscillations were similar in duration, amplitude, and frequency to those observed in other animal and human recordings, but uncorrelated with spiking. They likely arose from active ion channels in the axons, producing oscillations in the axonal membrane potential, but not from volume conduction. These spindle oscillations were independent of the thalamus with possible implications for function in cognitive consolidation separate from task and motor consolidation. The strongest oscillations were in the CA3 subregion sending axons both forward to the CA1 and back to the DG. Further studies of relative timing could show that calcium-mediated excitation in CA3 may send an inhibitory spindle signal back to DG that potentially clears and primes DG for the next input and forward spiking to CA1 to promote LTP together with other spiking inputs. These propositions are testable with pharmacological channel modulators and precise spatial–temporal dynamics in our system. Experiments with sleep drugs could have implications for sleep pathologies, treatments and side effects.

## Materials and methods

Data was collected from the experiments of Vakilna et al.^15^ using the four-compartment PDMS device with primary neurons connected through microfluidic tunnels over a 120 electrode array (MEA) (Fig. 1A). Original experiments were conducted in accordance with National Institutes of Health guidelines, with approval from the University of California, Irvine, Institutional Animal Care and Use Committee which follow the guidelines for the Care and Use of Laboratory Animals. Briefly, recordings used a Multichannel Systems MEA120 1100× amplifier (Multichannel Systems, Reutlingen, Germany). This study focused only on five minutes of spontaneous activity recordings, which were collected with the MC_Rack software at a sampling rate of 25 kHz at 37 °C for 5 min, in humidified 5% CO_2_, 9% O_2_, in culture medium. The recording began after three weeks of culture in NbActiv4 media (Thermo-Fisher, Carlsbad, CA). Arrays with poor growth or less than 80% active electrodes were rejected^15^. The four subregions contained neurons micro-dissected from the subregions of the hippocampal formation of postnatal day 4 Sprague–Dawley rats, namely the EC, DG, CA3 and CA1 + subiculum in order. Fifty-one microfluidic tunnels of 3 × 10 × 400 µm allowed axons to connect to adjacent compartments. Five of these tunnels per subregion were aligned over two 30 µm diameter electrodes that were 200 µm apart to measure the direction of action potential propagation. Recordings from nine arrays are reported.

### Spike and spindle detection

All the computations were performed using custom scripts written in MATLAB^®^ 2022a. Spikes were detected using the method discussed in Vakilna et al.^15^ with improved automation^36^. A fourth-order elliptic bandpass filter from 300 to 3000 Hz was applied to 5 min raw data sampled at 25 kHz. The filtered data was subjected to spike detection and sorting using Wave_Clus^37^. In order to adequately capture large spikes in tunnels, two thresholds were used for detection of the negative peak: 5–50 times standard deviation (SD] and 50.1 to 500 times SD. Previous analyses of these tunnel devices showed ~ 63% of tunnels contained only one axon^16^. Therefore, spikes could be combined into one cluster and axons could reliably be found after normalized matching indexing (NMI) between the two tunnel electrodes to find conduction time delays, and by extension, the direction of transmission. Peaks in the NMI histogram distribution of conduction times were then used to identify axons.

Spindle detection followed the filter methods of Silversmith et al.^19^, Kim et al.^17^ and Sela et al.^38^. The time series data recorded from the tunnels was downsampled from 25 to 1 kHz after applying a Kaiser window finite impulse response (FIR) anti-aliasing filter (50th order) in MATLAB^®^. Spindles were detected by applying an 8–16 Hz bandpass filter (6th-order high-pass and 8th-order low-pass Butterworth infinite impulse response (IIR) filter. Afterward, the envelope of the signal was extracted by computing the magnitude of the analytic signal from the Hilbert transform of the bandpass filtered signal. A time window was classified as a spindle if the upper envelope exceeded a threshold of 2.5 SD above the mean once and the lower envelope crossed a threshold of 1.5 SD below the mean for more than 300 ms. Multiple detections of the same spindle were eliminated by combining the spindles that were less than 500 ms apart^19,39^. Videos were created of the raw signal and the corresponding spindle-filtered signal using MATLAB Videowriter. The area under the curve, spindle duration, and inter-spindle interval were calculated for each spindle. The amplitude of each spindle was then calculated by dividing the area by the length. Phase locking of spikes with each spindle cycle was estimated by computing the polar histogram of phases and the phase angles of the spindle wave when the spike occurred. Time–frequency decomposition of the spindle was generated by computing continuous wavelet transform using an analytic Morse wavelet ($\upgamma$ = 3, ${p}^{2}$ = 60)^40^.

### Spindle nesting within slow waves

The method used to determine if spindles were evoked by slow oscillations was derived from Silversmith^19^. Slow oscillation epochs were defined as a positive-to-negative crossing with a peak to the left of the crossing and a trough to the right of the crossing. The epochs with the top 40% of peaks and top 40% of troughs were used. The time between the peak of the slow oscillation and the peak of the spindle was used to measure the degree of nesting between the two waves. The slow oscillation that directly preceded the spindle was used to measure the timing difference. We noticed that the timing differences between slow oscillations and spindles were log–log distributed. With log-binned histograms, a grid search was performed to find the short time boundary with the optimal R^2^ value between 0.001 s (the sampling rate) and 0.5 s.

### Slow wave and spike timing delays

In order to show that the spindles observed are not filter artifacts, the speed of LFP propagation along the axon was measured and compared to spike conduction delay. We calculated the spindle delay for the strongest spindles, the feedback axons from CA3 to DG and the feed-forward axons from CA3 to CA1. With the raw data, the MATLAB “findpeaks” function was employed to find the minima of the slow waves with a minimum peak-to-peak distance of 100 ms, a threshold exceeding 20% of the maximum negative amplitude of the LFP, a minimum threshold at − 100 μV, and a minimum peak width of 4 ms to exclude spikes. The minima were paired using the minimum time difference between peaks in upstream and downstream tunnel electrodes at a maximum 5 ms delay between channels. Any axon that had a standard error of conduction delays larger than its mean delay or a standard error of 0 was excluded. The distribution of delays was plotted for spikes and spindles across all single axons for each inter-region and direction from multiple arrays. From these distributions, a total mean and standard error were calculated. Differences in spike and slow wave conduction delays were evaluated by Student’s t-test, criterion *p* ≤ 0.05.

### Histogram of spike times with spindle phase

To correlate axon spikes with the phase of the spindles, polar histograms were generated for each subregion and feed direction (MATLAB^®^ Circular Statistics Toolbox). From the Hilbert transform of the 10–16 Hz filtered data, the phase angles within each spindle were distributed into 50 bins, for assignment and counting of spike times. A Hodges–Ajne test (omnibus test) for nonuniformity was performed for each subregion in each feed direction using the “circ_otest.m” function. The effect sizes were then calculated by subtracting the largest bin count from the with the smallest bin count, divided by the standard deviation from the nine arrays.

### Statistics

Each spindle quantity was fitted to a log-normal distribution and the parameters were estimated by fitting Gaussian distribution to the log-transformed histogram. Mean and standard deviations were computed for each histogram. Recordings with only one spindle oscillation in 5 min or spindles less than 0.4 s length were not included. To measure oscillatory relationships of waveforms from two recording sites, the non-spindle regions of the recording were set to zero. Then, Pearson correlations (r) were calculated using the xcorr function of MATLAB^®^ normalized from 0 to 1 for the entire five minute recording and unscaled for other subregions. The significance of difference in means were estimated by computing one-way analysis of variance (ANOVA), followed by Tukey’s HSD test.

### Significance

Spindle oscillations contribute to the consolidation of memory events in the mammalian brain, but their origin and route of transmission, particularly in the hippocampus is unclear. To address this gap, we reverse engineered the rat hippocampus into four subregions over an electrode array without thalamic neurons. The MEMS device confined single inter-regional axons into microfluidic channels between entorhinal, dentate, CA3 and CA1 neurons. Surprisingly, spontaneous spindle oscillations of 10–16 Hz were found on these communicating axons, propagating at slower velocities than spikes, and strongest in feedback from the CA3 to the DG. Oscillations in axon membrane potential within the hippocampal formation provide a distinct form of transmission, independent of the thalamus and volume conduction of local field potentials.

### Supplementary Information

Supplementary Video 1.

Supplementary Video 2.

Supplementary Information 1.

## Data Availability

Spike and oscillation time data is available at https://datadryad.org/stash/dataset/doi:10.5061/dryad.fqz612jzz. Scripts are available for our preprocessing pipeline: https://github.com/Brewer-Neurolab/Lassers-2023-Axon-Flow, and Spindle analysis: https://github.com/Brewer-Neurolab/Wang-Spindles-in-Axons.

## References

[1] Loomis AL, Harvey EN, Hobart G (1935). Potential rhythms of the cerebral cortex during sleep. Science.

[2] Fernandez LMJ, Luthi A (2020). Sleep spindles: Mechanisms and functions. Physiol. Rev..

[3] Ketz N, Jones AP, Bryant NB, Clark VP, Pilly PK (2018). Closed-loop slow-wave tACS improves sleep-dependent long-term memory generalization by modulating endogenous oscillations. J. Neurosci.: Off. J. Soc. Neurosci..

[4] Hanert A, Weber FD, Pedersen A, Born J, Bartsch T (2017). Sleep in humans stabilizes pattern separation performance. J. Neurosci.: Off. J. Soc. Neurosci..

[5] Geva-Sagiv M (2023). Augmenting hippocampal-prefrontal neuronal synchrony during sleep enhances memory consolidation in humans. Nat. Neurosci..

[6] Andrillon T (2011). Sleep spindles in humans: insights from intracranial EEG and unit recordings. J. Neurosci.: Off. J. Soc. Neurosci..

[7] Pinault D (2004). The thalamic reticular nucleus: structure, function and concept. Brain Res. Brain Res. Rev..

[8] Steriade M, Domich L, Oakson G, Deschenes M (1987). The deafferented reticular thalamic nucleus generates spindle rhythmicity. J. Neurophysiol..

[9] Steriade M, McCormick DA, Sejnowski TJ (1993). Thalamocortical oscillations in the sleeping and aroused brain. Science.

[10] Sullivan D, Mizuseki K, Sorgi A, Buzsaki G (2014). Comparison of sleep spindles and theta oscillations in the hippocampus. J. Neurosci.: Off. J. Soc. Neurosci..

[11] Andrade KC (2011). Sleep spindles and hippocampal functional connectivity in human NREM sleep. J. Neurosci..

[12] Ngo HV, Fell J, Staresina B (2020). Sleep spindles mediate hippocampal-neocortical coupling during long-duration ripples. Elife.

[13] Helfrich RF (2019). Bidirectional prefrontal-hippocampal dynamics organize information transfer during sleep in humans. Nat. Commun..

[14] Cox R, Ruber T, Staresina BP, Fell J (2020). Phase-based coordination of hippocampal and neocortical oscillations during human sleep. Commun. Biol..

[15] Vakilna YS, Tang WC, Wheeler BC, Brewer GJ (2021). The flow of axonal information among hippocampal subregions: 1. Feed-forward and feedback network spatial dynamics underpinning emergent information processing. Front. Neural Circuits.

[16] Narula U (2017). Narrow microtunnel technology for the isolation and precise identification of axonal communication among distinct hippocampal subregion networks. PLoS ONE.

[17] Kim J, Gulati T, Ganguly K (2019). Competing roles of slow oscillations and delta waves in memory consolidation versus forgetting. Cell.

[18] Gonzalez C, Jiang X, Gonzalez-Martinez J, Halgren E (2022). Human spindle variability. J. Neurosci.: Off. J. Soc. Neurosci..

[19] Silversmith DB, Lemke SM, Egert D, Berke JD, Ganguly K (2020). The degree of nesting between spindles and slow oscillations modulates neural synchrony. J. Neurosci.: Off. J. Soc. Neurosci..

[20] Panas D, Malinowska U, Piotrowski T, Zygierewicz J, Suffczynski P (2013). Statistical analysis of sleep spindle occurrences. PLoS ONE.

[21] Tang W, Shin JD, Frank LM, Jadhav SP (2017). Hippocampal-prefrontal reactivation during learning is stronger in awake compared with sleep states. J. Neurosci..

[22] Vyleta NP, Borges-Merjane C, Jonas P (2016). Plasticity-dependent, full detonation at hippocampal mossy fiber-CA3 pyramidal neuron synapses. eLife.

[23] Oyanedel CN (2014). Role of slow oscillatory activity and slow wave sleep in consolidation of episodic-like memory in rats. Behav. Brain Res..

[24] Nam Y, Wheeler BC (2011). In vitro microelectrode array technology and neural recordings. Crit. Rev. Biomed. Eng..

[25] Hagen E (2017). Focal local field potential signature of the single-axon monosynaptic thalamocortical connection. J. Neurosci..

[26] Steriade M, Dossi RC, Nunez A (1991). Network modulation of a slow intrinsic oscillation of cat thalamocortical neurons implicated in sleep delta waves: cortically induced synchronization and brainstem cholinergic suppression. J. Neurosci..

[27] Gooch HM (2022). High-fidelity dendritic sodium spike generation in human layer 2/3 neocortical pyramidal neurons. Cell reports.

[28] Traub RD, Miles R, Wong RK (1989). Model of the origin of rhythmic population oscillations in the hippocampal slice. Science.

[29] Halgren AS, Siegel Z, Golden R, Bazhenov M (2023). Multielectrode cortical stimulation selectively induces unidirectional wave propagation of excitatory neuronal activity in biophysical neural model. J. Neurosci.: Off. J. Soc. Neurosci..

[30] Sun JJ, Luhmann HJ (2007). Spatio-temporal dynamics of oscillatory network activity in the neonatal mouse cerebral cortex. Eur. J. Neurosci..

[31] Peyrache A, Battaglia FP, Destexhe A (2011). Inhibition recruitment in prefrontal cortex during sleep spindles and gating of hippocampal inputs. Proc. Natl. Acad. Sci. U. S. A..

[32] Boutin A (2018). Transient synchronization of hippocampo-striato-thalamo-cortical networks during sleep spindle oscillations induces motor memory consolidation. Neuroimage.

[33] Gulati T, Guo L, Ramanathan DS, Bodepudi A, Ganguly K (2017). Neural reactivations during sleep determine network credit assignment. Nat. Neurosci..

[34] Klinzing JG, Niethard N, Born J (2019). Mechanisms of systems memory consolidation during sleep. Nat. Neurosci..

[35] Varela C, Kumar S, Yang JY, Wilson MA (2014). Anatomical substrates for direct interactions between hippocampus, medial prefrontal cortex, and the thalamic nucleus reuniens. Brain Struct. Funct..

[36] Lassers SB, Vakilna YS, Tang WC, Brewer GJ (2023). The flow of axonal information among hippocampal subregions: 2. Patterned stimulation sharpens routing of information transmission sharpens routing of information transmission. Front. Neural Circuits.

[37] Chaure FJ, Rey HG, Quian Quiroga R (2018). A novel and fully automatic spike-sorting implementation with variable number of features. J. Neurophysiol..

[38] Sela Y, Vyazovskiy VV, Cirelli C, Tononi G, Nir Y (2016). Responses in rat core auditory cortex are preserved during sleep spindle oscillations. Sleep.

[39] Schimicek P, Zeitlhofer J, Anderer P, Saletu B (1994). Automatic sleep-spindle detection procedure: aspects of reliability and validity. Clin. Electroencephalogr..

[40] Lilly JM (2017). Element analysis: a wavelet-based method for analysing time-localized events in noisy time series. Proc. Math. Phys. Eng. Sci..

