# Supplementary material for: Spindle oscillations in communicating axons within a reconstituted hippocampal formation are strongest in CA3 without thalamus
Source: Sci Rep. 2024 Apr 10;14:8384. doi: 10.1038/s41598-024-58002-0 (PMC11006914; doi:10.1038/s41598-024-58002-0)
Supplement: Supplementary file 3 — Supplementary Information 1. [file 41598_2024_58002_MOESM3_ESM.docx]

[
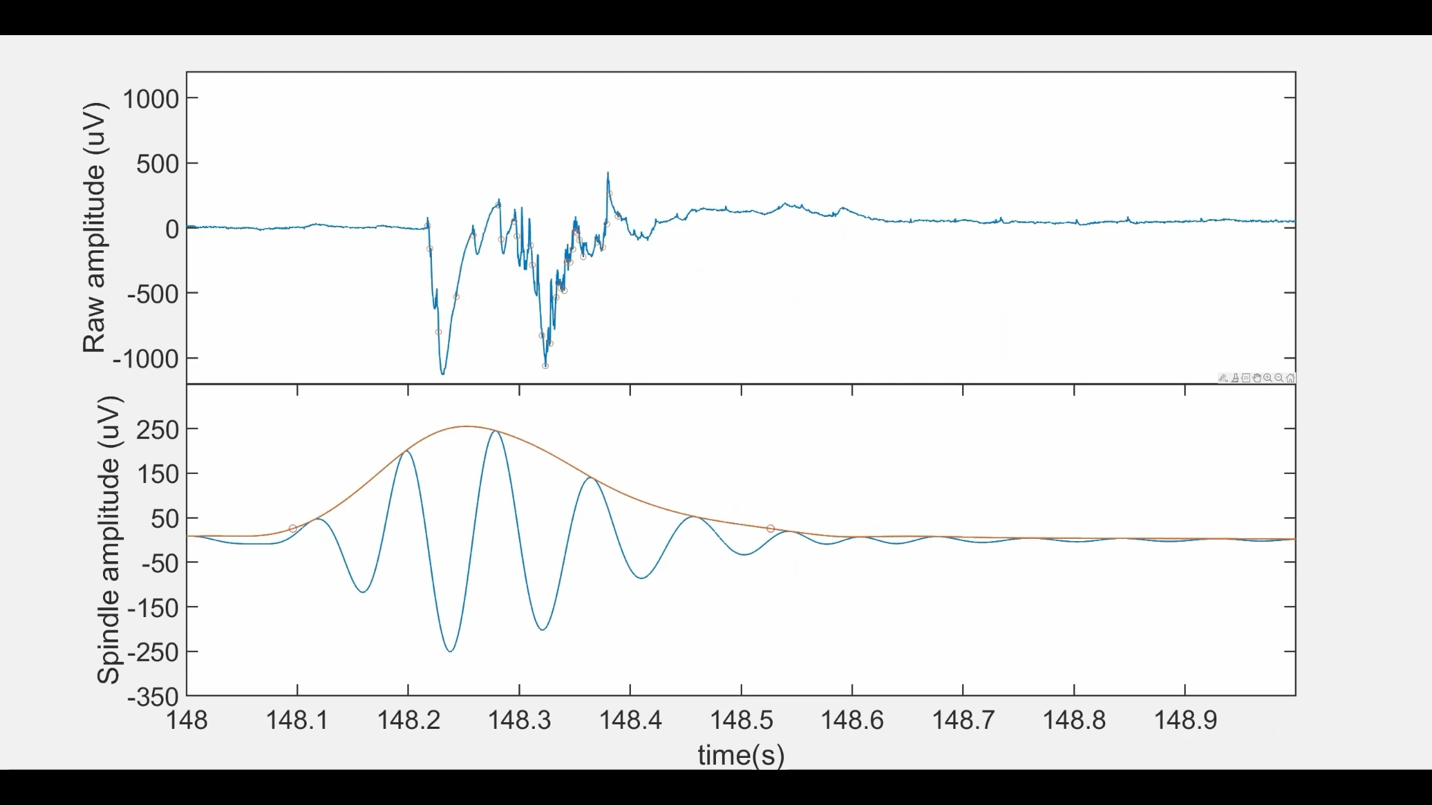
](file:////Users/gregoryjbrewer/Desktop/manuscriptsM/2023%20Mengke%20Wang%20Spindles/F10-G10%2040s%20DG-CA3%20FB%2040s%20240308.mp4)[
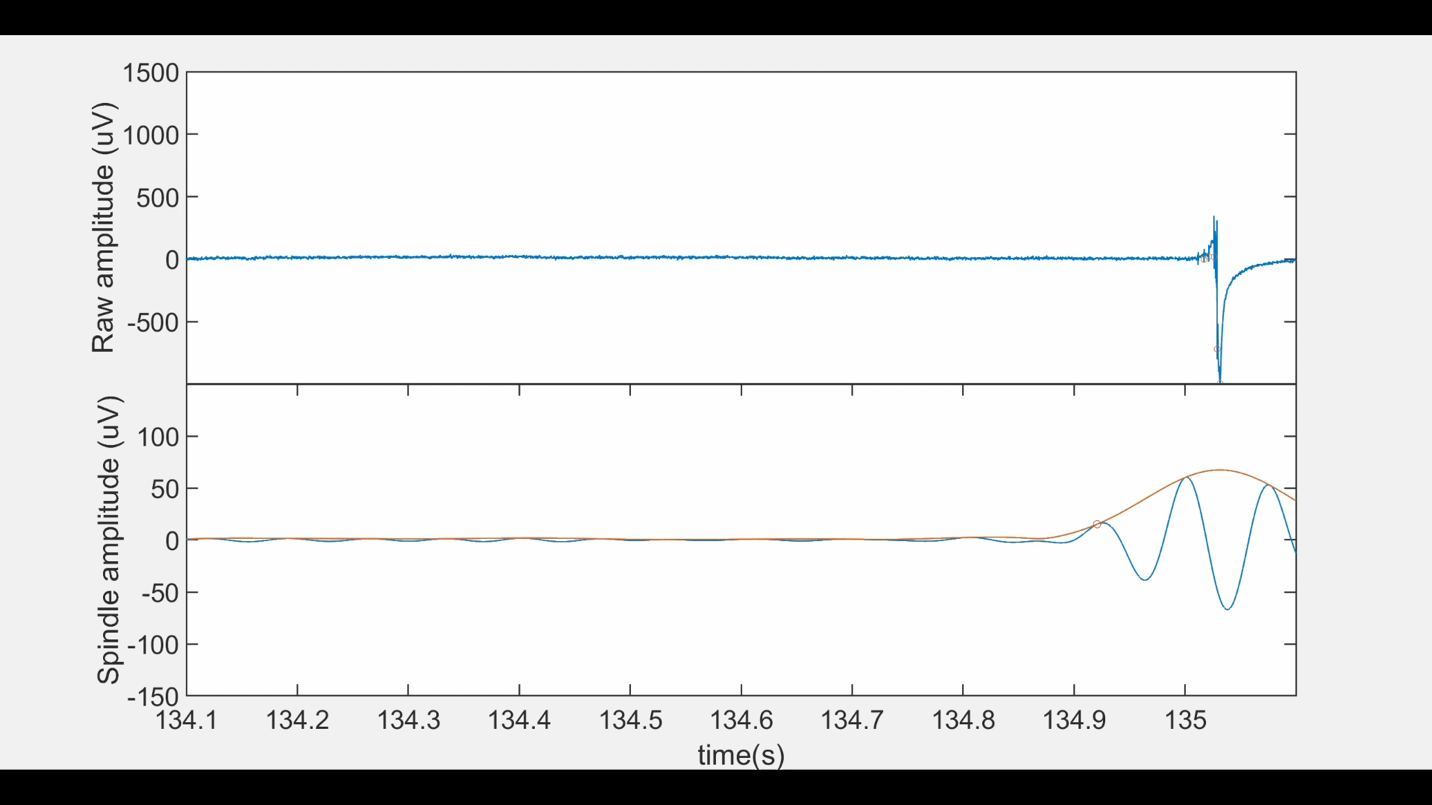
](file:////Users/gregoryjbrewer/Desktop/manuscriptsM/2023%20Mengke%20Wang%20Spindles/M7%20M6%2040s%20CA3-CA1%20FF%20240301.mp4)Supplementary figures, Wang et al.

A) CA3-CA1 feedforward spindles (M7-M6)

B) CA3 to DG feedback spindles (F10-G10)

Supplementary Figure 1. Spindle videos. Top panel, raw data, including spikes marked at their peak (o). Bottom panel, spindle oscillation from 10-16 Hz filter. Start and end of spindle marked by orange (o). Orange trace marks spindle envelope.

**
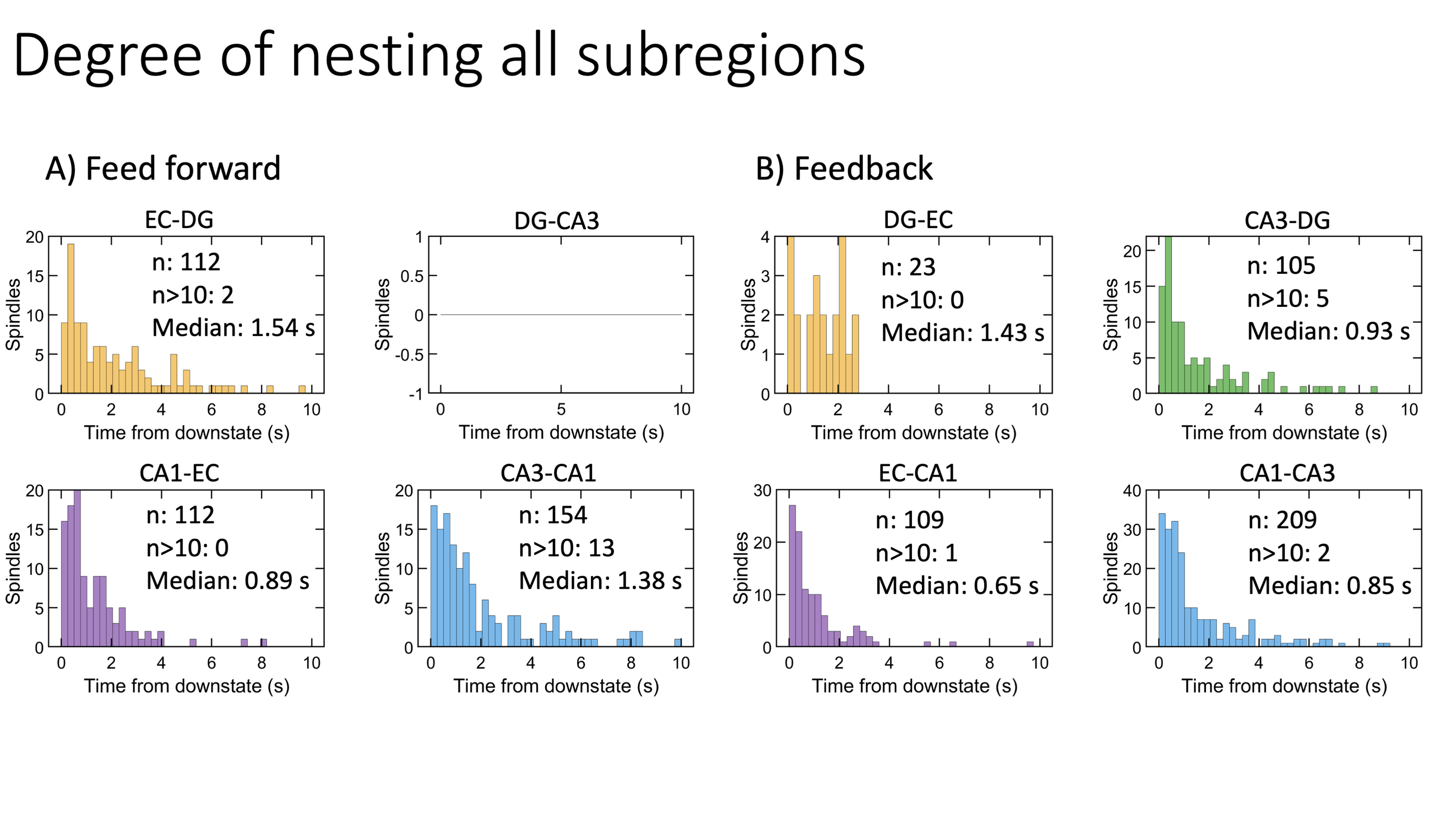
Supplementary Fig. 3.** Distributions of spindles in time from the onset of a slow wave by subregions. A) feed forward axons, and B) feedback axons


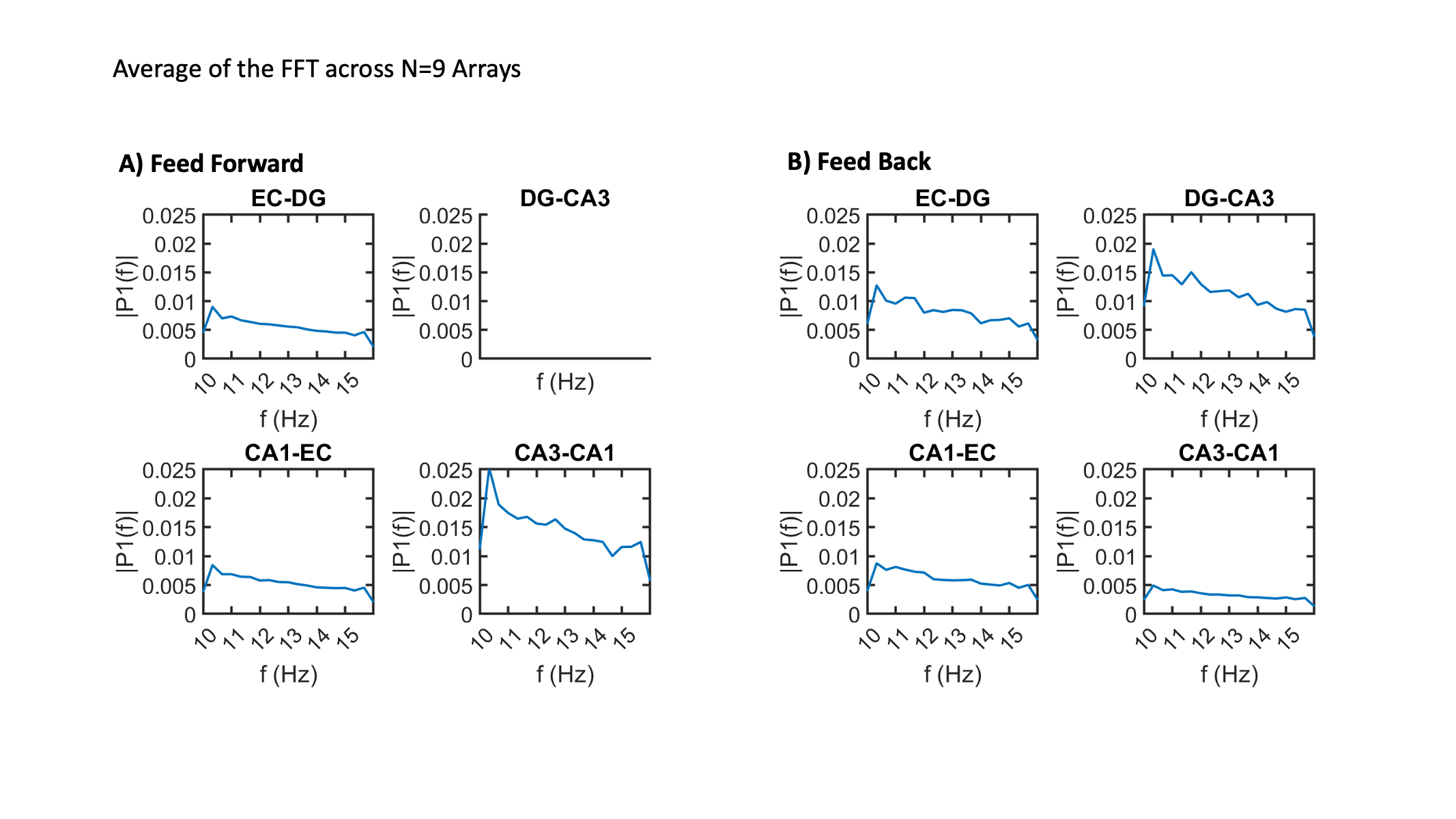
**Supplementary Fig. 2.** 10-12 Hz peaks in power distributions of fast Fourier transforms within the 10-16 Hz range of spindle frequencies from nine arrays by subregion and A) feed forward axons, and B) feedback axons.

**
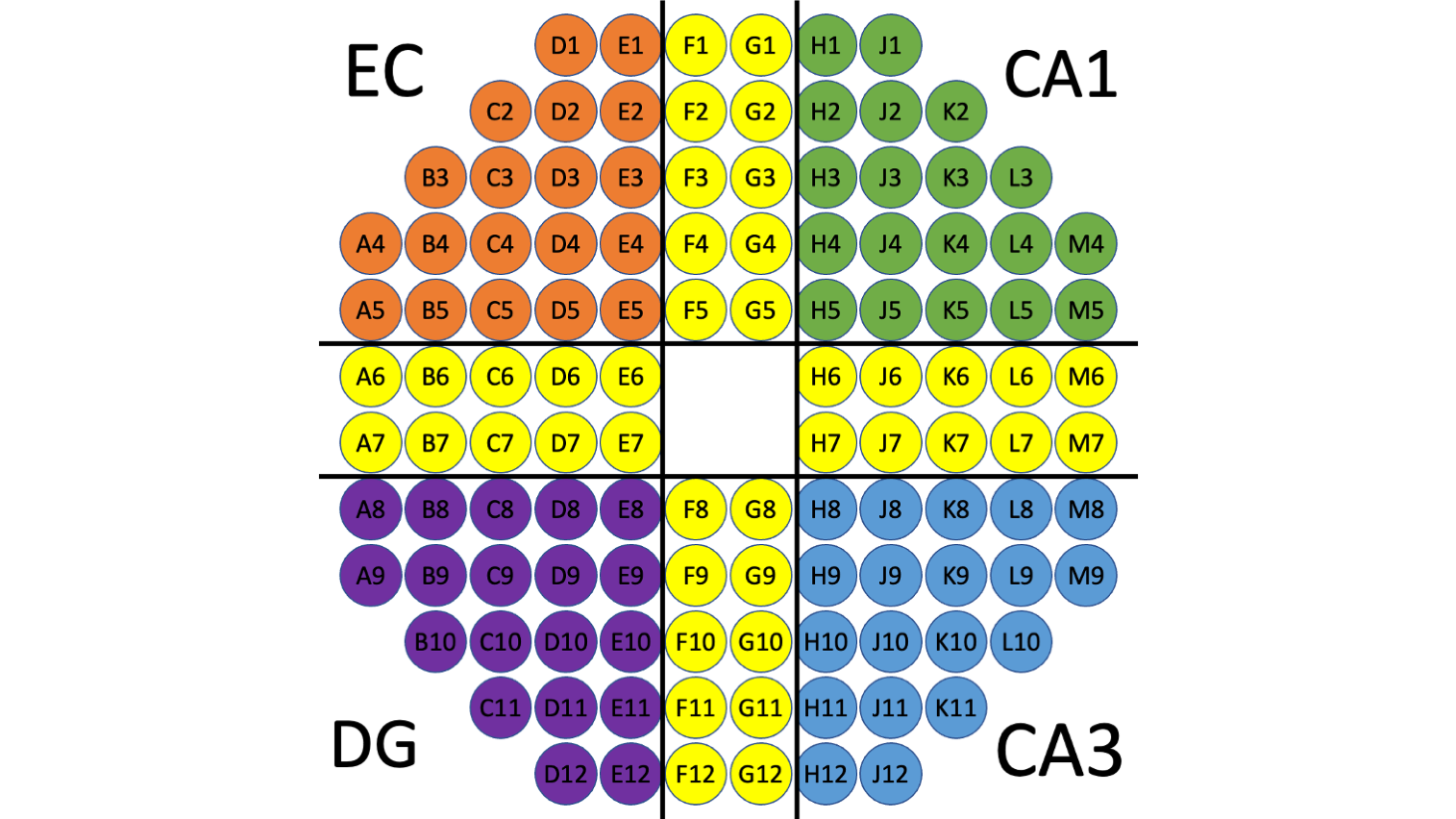
Supplementary Fig. 4.** Electrode names in each subregional compartment and their interconnecting axon tunnels (yellow). Half of recordings had this counter-clockwise order reversed with CA1 and DG swapped.
